# Microglial contribution to the pathology of neurodevelopmental disorders in humans

**DOI:** 10.1007/s00401-023-02629-2

**Published:** 2023-09-01

**Authors:** Rugile Matuleviciute, Elizabeth T. Akinluyi, Tim A. O. Muntslag, Jennifer M. Dewing, Katherine R. Long, Anthony C. Vernon, Marie-Eve Tremblay, David A. Menassa

**Affiliations:** 1 MRC Centre for Neurodevelopmental Disorders, King’s College London, London, UK; 2 Department of Basic and Clinical Neuroscience, Institute of Psychiatry, Psychology and Neuroscience, King’s College London, London, UK; 3 Division of Medical Sciences, University of Victoria, Victoria, Canada; 4 Department of Pharmacology and Therapeutics, Afe Babalola University, Ado Ekiti, Nigeria; 5 Princess Maxima Centre for Paediatric Oncology, Utrecht, The Netherlands; 6 Faculty of Medicine, University of Southampton, Southampton, UK; 7 Centre for Developmental Neurobiology, Institute of Psychiatry, Psychology and Neuroscience, King’s College London, London, UK; 8 Department of Neuropathology & The Queen’s College, University of Oxford, Oxford, UK; 9 Department of Women’s and Children’s Health, Karolinska Institutet, Solna, Sweden

**Keywords:** Human microglia, Neurodevelopmental disorders, Autism spectrum conditions, Schizophrenia, Spatial transcriptomics, Neurodevelopmental models, Human-induced pluripotent stem cells

## Abstract

Microglia are the brain’s resident macrophages, which guide various developmental processes crucial for brain maturation, activity, and plasticity. Microglial progenitors enter the telencephalic wall by the 4th postconceptional week and colonise the fetal brain in a manner that spatiotemporally tracks key neurodevelopmental processes in humans. However, much of what we know about how microglia shape neurodevelopment comes from rodent studies. Multiple differences exist between human and rodent microglia warranting further focus on the human condition, particularly as microglia are emerging as critically involved in the pathological signature of various cognitive and neurodevelopmental disorders. In this article, we review the evidence supporting microglial involvement in basic neurodevelopmental processes by focusing on the human species. We next concur on the neuropathological evidence demonstrating whether and how microglia contribute to the aetiology of two neurodevelopmental disorders: autism spectrum conditions and schizophrenia. Next, we highlight how recent technologies have revolutionised our understanding of microglial biology with a focus on how these tools can help us elucidate at unprecedented resolution the links between microglia and neurodevelopmental disorders. We conclude by reviewing which current treatment approaches have shown most promise towards targeting microglia in neurodevelopmental disorders and suggest novel avenues for future consideration.

## Introduction

Neurodevelopmental disorders (NDDs) are a group of complex conditions, with an onset either early during childhood or later during adolescence. They result in motor, sensory, and cognitive impairments of varying severity across the lifespan [130, 192]. The term ‘neurodevelopmental’ has been applied broadly to encompass a set of clinically and causally different conditions, notably with neurological and psychiatric presentations such as autism spectrum conditions (ASCs) and schizophrenia (SZ) [134, 192]. ASCs and SZ are common NDDs that account for a significant disease burden, according to a UK-based population study [72]. This makes ASCs and SZ a natural starting point when investigating cellular and molecular mechanisms underlying NDD pathology.

ASCs manifest in early childhood, typically presenting before the age of 3 years [72]. The median prevalence of ASCs is 1 in 100 and ASCs are 4 times more prevalent in males than females [215]. Diagnostic criteria for ASCs include persistent deficits in social communication and interaction, repetitive behaviours and special interests [4]. SZ also affects around 1 in 100 people worldwide [78]. While traditionally SZ was thought to affect both sexes equally, a meta-analysis suggested a slightly greater risk in men [85]. The key symptoms of this condition are classified as positive, negative, and cognitive [78]. SZ first manifests in late adolescence and early adulthood, with negative symptoms usually first to appear [78]. While ASCs and SZ are highly heterogenous conditions, there is overlap between their pathophysiological mechanisms specifically those involving the immune system.

In this context, changes in the innate immune system involving microglial cells have been consistently reported in ASCs and SZ [34, 64, 118, 125]. Circulating cytokine profiles are altered in patients with ASCs and SZ [37, 220]. Increased levels of interleukins- (IL-) 6 and IL-8 have been measured in the cerebrospinal fluid (CSF) of SZ patients [48]. Molecules linked with inflammation and immune function, including IL-8, as well as immunoglobulin A (IgA), IL-13 and macrophage migration inhibitory factor (MIF) amongst others, have been additionally suggested as potential plasma biomarkers for SZ [18, 35]. Changes to the cytokine landscape in NDDs may, in part, be shaped by environmental exposures prenatally and during early life. Indeed, a meta-analysis revealed that viral childhood infections are associated with an increased risk of psychotic disorders into adulthood [90]. Similarly, mothers admitted to hospital, particularly with bacterial infections, are at a greater risk of delivering a child later diagnosed with an ASC [216]. Multiple infections during pregnancy have also been associated with an increased ASC risk in the offspring [216]. Immune contributions to ASCs and SZ have been further noted on a genetic level. The major histocompatibility complex (MHC) region includes a number of loci associated with SZ that were identified through genome-wide association studies (GWAS) [162, 163]. Amongst those, variants of the complement component 4 (*C4*) genes are particularly notable [171]. Similarly, in the case of ASCs, associations between variants in human leukocyte antigen (*HLA*) genes and the condition have been identified [196].

Overall, the immune system and NDDs are tightly connected. Microglia which are components of the brain’s innate immunity, are inherent to the pathological signature of NDDs. However, it remains unclear whether microglia directly alter neurodevelopmental trajectories leading to NDDs and/or if their effects are reactive to a causative insult. Critically, much of our knowledge of microglia in typical and atypical brain development derives from rodent studies even though there are marked differences between human and rodent microglia. Furthermore, cognitive impairments in NDDs such as SZ and ASCs involving language, thought processing, memory and executive deficits are characteristically human [122, 182]. In this article, we first discuss how microglia shape basic neurodevelopmental processes by focusing on human-based findings. We then review the state of affairs and concur on the role of microglia in two main disorders along the neurodevelopmental continuum: ASCs and SZ. Finally, we review recent technological advances that have helped develop our understanding of microglial biology and discuss some promising avenues for future interventions targeting microglia in NDDs.

### Microglial functions during development

The development of neural circuits in the central nervous system (CNS) requires the involvement of all its neuronal and non-neuronal cells. Amongst these, microglia—the resident macrophages of the CNS—play a crucial role in mediating optimal brain development, maturation and functioning [145, 179]. While most of the work on microglial functions in the developing brain comes from rodent studies, we primarily focus here on human-based findings.

Microglia originate from extraembryonic yolk sac progenitors that colonise the neuroepithelium of the human forebrain from the 4th postconceptional week (pcw) onwards [119, 121, 204]. Microglial progenitors begin to proliferate as soon as they arrive to the developing forebrain and as early as the 4th pcw [121]. By the 9th pcw, they become immunocompetent, meaning that the cells acquire the ability to adeptly recognise and respond to immune-associated stimuli [94].

Microglia coexist in multiple functional and morphological states throughout different stages of CNS development, transitioning from amoeboid to intermediate rod-like, and eventually becoming ramified [121]. These varying morphologies likely correspond to specific functions notably associated with the neuroanatomical process or compartment they inhabit or attempt to colonise, the stage of life (development, adulthood, or ageing), the sex of the individual, and the challenges they encounter (disease, injury, etc.) across life, as revealed by morphological, ultrastructural, epigenetic, transcriptomic, metabolomic, and proteomic data [9, 111, 120, 121, 149, 161]. For instance, intermediate rod-shaped microglia are observed in white matter tracts, such as the corpus callosum and the external capsule, towards the end of the 30th pcw where they suggestively participate in axonal guidance, synaptogenesis, and neurodevelopmental apoptosis [204]. Single-cell RNA sequencing (scRNA-seq) of fluorescence-activated cell sorting (FACS)-sorted human microglia obtained from fetuses between the 7th and 16th pcw following elective pregnancy terminations, revealed a cluster of proliferative-region-associated microglia (PAM) which share some transcriptional signature overlap with phagocytic disease-associated microglia (DAM) and neurodegenerative disease microglia (MGnD) [94]. These microglial cells express multiple gene markers such as apolipoprotein E (*APOE*), and cluster of differentiation 68 (*CD68*), which are associated with the DAM and MGnD states [88, 94, 95].

**Fig. 1:**
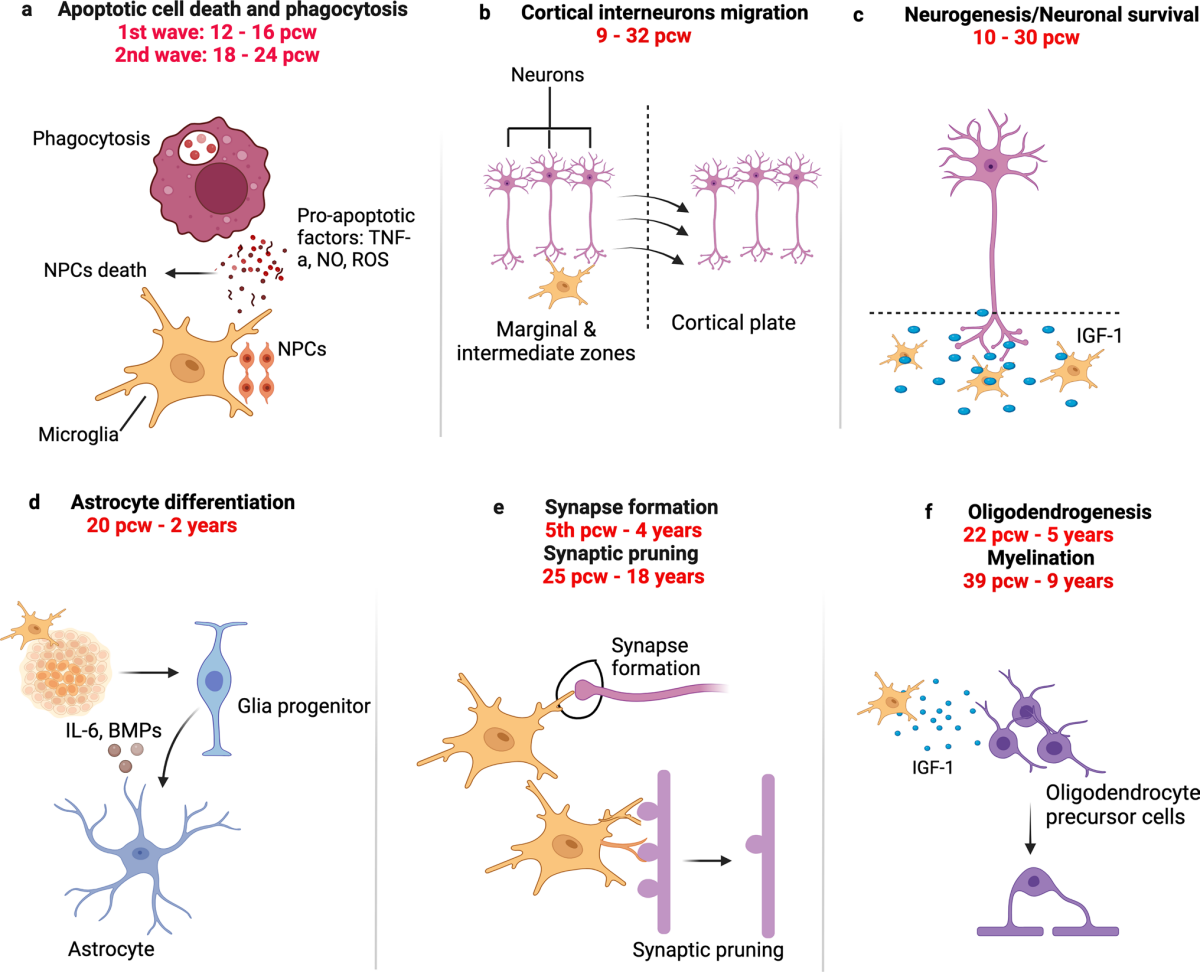
Main functions of microglia in the developing human CNS. **a** Microglia regulate the number of neuronal cells, notably by engaging in active phagocytosis of progenitor cells and promoting the apoptosis of differentiated cells [17, 158]. **b** Microglia regulate neuronal migratory processes within the neocortex [17]. **c** Microglia also release factors (such as insulin growth factor 1 (IGF-1), that support the growth and survival of neurons [17]. **d** Microglia impact onto astrocyte differentiation via direct cell-to-cell communication, secretion of microglia-derived and trophic factors [5, 135]. **e** Microglia mediate synapse elimination for instance via the engulfment of presynaptic inputs [119, 121]. **f** Microglia promote the proliferation and differentiation of oligodendrocyte precursor cells, and they interact with oligodendrocytes to mediate their maturation and survival, as well as contribute to maintaining the myelination status of the CNS [77]

Microglia contribute to typical CNS development, and these cells play several roles during the prenatal and early postnatal stages of development and maturation. Rodent studies have shown that microglia participate in neurogenesis, oligodendrogenesis [62, 115, 174], astrogliogenesis [110], axonal myelination [136], as well as synapse formation [123], maturation and pruning [145] via specialized interactions with neuronal and non-neuronal cells (Fig. 1). Although studies involving human microglia can be complex, research on chimeric models that involve transplanting human-induced pluripotent stem cells (hiPSCs)-derived microglial progenitor cells [41, 66] or microglia [107] into the mouse brain, human brain organoids [41], and embryonic and fetal tissues [149] have confirmed previous findings in animal models. In humans, microglia have been linked with crucial developmental processes such as neurogenesis, programmed cell death and apoptotic cell clearance, neuronal migration, white matter tract formation, and synaptic pruning [119]. The subsequent subsections outline the roles of microglia in shaping the developing human brain.

*Neurogenesis and neuronal migration.* Insights into the involvement of human microglia in developmental neurogenesis have been gleaned from various studies. These cells have been linked with neurogenesis from the 8th pcw to the 22nd pcw and neural migration from the 8th pcw to the 30th pcw [17]. Popova et al*.* engrafted primary human fetal ionised calcium-binding adapter molecule 1 (IBA1) positive microglia from *post-mortem* cortical tissue into cerebral organoids [155]. By using CD68 staining, they showed that prenatal microglia exhibited active phagocytosis of progenitor cells and synapses in the organoids, thereby modulating synaptic density and regulating the production and maturation of new neurons [155]. Also, in an in vitro electrophysiological study which aimed to evaluate neuronal functions at multiple developmental time points, microglia-like cells derived from hiPSCs expedited neuronal maturation by regulating the development of single-cell sodium and potassium currents, which ultimately manifested as increased neuronal network activity in human cerebral organoids [21].

*Developmental apoptosis.* During the early developmental processes of neuronal proliferation, migration and differentiation, neuronal precursors and neurons that are less active and fail to establish appropriate synaptic connections undergo apoptosis [159]. In the developing human telencephalon, microglia are associated with early embryonic apoptosis and apoptotic cell clearance from the 12^th^ pcw to the 16^th^ pcw [158]. This close interaction between microglia and neuronal precursors provides support for their involvement in the formation of the telencephalon in the prenatal stages. Microglial clusters expressing *CD68* [2], *IBA1*, *CD45*, and the lectin, Ricinus Communis agglutinin I (*RCA-1*) [126, 127], have been found to accumulate in the cortical subplate and future basal ganglia of *post-mortem* human fetal tissues. It is speculated that these clusters contribute to neurodevelopmental apoptosis, as well as axonal guidance, and synaptogenesis [204].

*Synapse formation and maturation.* In humans, microglial cells likely participate in early cortical synaptogenesis which occurs in Cajal-Retzius cells, a group of early neurons crucial for cortical development, as early as the 5th pcw and the presubplate and preplate neurons as early as the 8.5th pcw [92, 119, 121]. However, direct evidence about microglial involvement in synapse formation in humans is lacking.

*Cortical folding.* In addition to shaping the developing brain on a cellular and circuit level, there is increasing evidence that microglia may also contribute on a tissue level. There is already some direct evidence for a change in cortical structure linked to microglial activities. In humans, microglia are found in transient cortical structures during fetal brain development, such as the subplate, between 12th and 13th pcw [120]. It is also hypothesized that the small changes in cell shape that they mediate, such as progenitor cell engulfment [158] and synaptic pruning and wiring [119], when combined, can lead to much larger changes in cortical organization and consequently cortical shape.

A key feature of human fetal brain development is the generation of the cortical folds, the gyri, and sulci, during the second half of gestation. This morphological change occurs alongside several critical developmental events, including neuronal migration, gliogenesis and an increase in microglial numbers, all of which are thought to occur before 26 pcw [186]. This influx of microglia has been proposed to aid the cortical expansion required for the generation of cortical folds, in particular their tangential migration in the developing cortex [16]. Another possible mechanism for microglial contribution to cortical folding is their regulation of basal progenitors, the neural progenitors thought to be responsible for the increased number of neural cells seen in gyrified species. Microglia were recently linked to a reduction in cell stress in these basal progenitors [155], which may facilitate their proliferative capacity and the generation of increased numbers of neurons required for cortical expansion and folding. Further indication of the role of microglia in cortical size and shape can be found in patients with cortical malformations. Focal clusters of microglia were found in patients with Walker Warburg Syndrome, a rare genetic disorder, characterised by muscular dystrophy and brain malformations, and were associated with areas of type II lissencephaly, characterised by a smooth brain surface with cobblestone-like irregularites, in the cortical plate [199]. Reactive microglia are also localized in areas of cortical dysplasias [13] and clustered in areas of polymicrogyria in patients with Cytomegalovirus (CMV) infections [180]. Understanding their role in regulating typical development and cortical folding, and how this relates to their inflammatory role in cortical malformations, will be important. This is especially relevant for understanding their role in NDDs discussed below as some patients will have cortical malformations and microglial dysfunction as part of their disorder as discussed later in this review.

Overall, microglia are dynamic cells that participate in various processes in the developing brain. Considerable differences between humans and rodents exist highlighting the shortcomings in the translatability of microglial findings to human neurodevelopment. We discuss these differences below.

### Why do we need to focus on human microglia?

Compared to mouse microglia, human microglia are more complex morphologically and display reduced proliferation rates, as well as specific differences in genes regulating critical endogenous processes like inflammation and the cell cycle [144, 174]. A study analysing microglial heterogeneity across species at different stages of life, reveals that the human brain has a larger microglial cell volume and a lower neuron-to-microglia ratio than the mouse brain. Moreover, higher microglial density is observed in the frontal cortex of the mouse compared to the human, whereas microglial density in the human hippocampus, cerebellum, and white matter is higher than in the mouse among corresponding regions [51]. In a study employing scRNA-seq on FACS-purified microglia, significant overlaps were found between the transcriptome profiles of human fetal microglia (gestational age between 12 and 22 pcws) and mouse fetal microglia (E18.5), when investigating the influence of sex and the microbiome on microglia. However, the study specifically highlighted the absence of sexual dimorphism in the human *versus* mouse transcriptomic signatures during mid-gestation [193]. Contrary to the observed age-dependent sex differences in the context of normal neurodevelopment, in rodents [169], a recent study employed a combination of techniques, including histology, advanced imaging, and three-dimensional reconstruction, to investigate microglial populations in *post-mortem* embryonic and fetal brain tissues, using IBA1, transmembrane protein 119 (TMEM119), purinergic receptor P2Y12 (P2RY12), and C-X3-C motif chemokine receptor 1 (CX3CR1) markers to assess and characterise microglial properties, reporting an extremely low or absent sexual dimorphism of microglial density, morphology and migrating capacity across human cortical development [121].

To further emphasize the importance of considering species-specific factors in understanding microglial development, a recent study employed principal component analysis and batch effect verification techniques to comprehensively analyze the developmental transcriptomes of human and mouse microglia [213]. The study investigated potential species-specific differences in microglial maturation, highlighting the significance of species-related factors in this process. The researchers utilized mouse microglia samples at E14.5, early post-natal stages P4/P5, a juvenile stage (P30), and adulthood (P100), alongside human microglia samples representing prenatal, pediatric, adolescent, and adult stages. The findings revealed distinct expression profiles between prenatal and post-natal microglia, and interestingly, E14.5 and P4/P5 mouse microglia exhibited similarities with human prenatal cells. Notably, in mouse pups, microglia formed a separate cluster distinct from their juvenile and adult counterparts, whereas human pediatric microglia were closely grouped with adult and adolescent samples. Based on the observed discrepancy, the researchers posited the hypothesis that mouse pediatric microglia might experience a developmental delay in maturation relative to their human counterparts [213].

These observations highlight the importance of studying human or humanised models to improve our understanding of the roles that microglia play in human conditions such as ASCs and SZ amongst other NDDs.

### Evidence for microglial involvement in ASCs

ASCs are a heterogeneous group of conditions, diagnosed based on symptoms in two core domains: the social communication impairments’ domain and the stereotypical behaviours and restricted interests’ domain [4]. ASCs rarely present on their own and often, affected individuals have sensory anomalies in the visual, olfactory, auditory, and somatosensory domains as well as comorbidities such as epilepsy and sleeping disorders [4]. There is no cure for ASCs, and early diagnosis is important to identify a treatment plan for comorbidities (e.g., epilepsy medication, ADHD medication, etc.), a care plan and a suitable environment/school for affected children to develop and thrive. ASCs are largely genetic but maternal factors can increase the risk of an ASC in the baby in a subset of cases. Factors can be immune, teratogenic, and infectious. Altogether, ASCs represent a complex set of disorders with a wide range of severities which remain challenging to study.

**Fig. 2:**
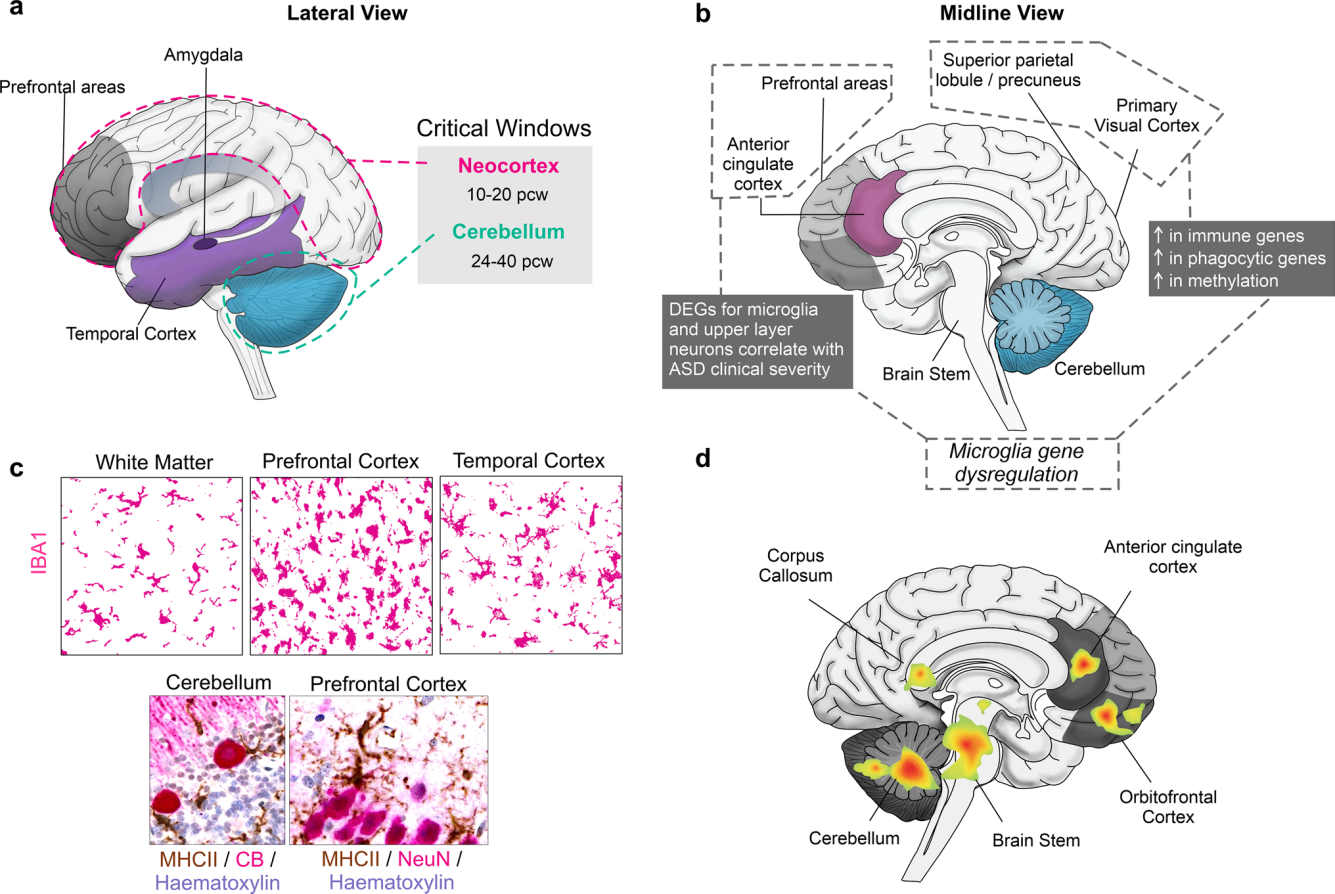
Microglial findings in ASCs. **a** Lateral view of the human brain with the critical/sensitive windows for ASC development, which include the cerebellum and the neocortex [209]. **b** Microglial gene dysregulation observed in ASC samples from the prefrontal and cingulate [203] as well as the primary visual areas and the superior parietal lobule [49]. **c** Microglial densities (IBA1^+^, magenta) are unchanged, higher, or lower in these brain areas compared to typically developing controls (top panel). In the cerebellum and neocortex, neuroinflammatory processes are heightened and microglia express MHC-II (brown) and cup Purkinje Calbindin^+^ cells in the cerebellum (CB^+^, magenta) and neurons in the neocortex (NeuN^+^, magenta) (bottom panel). Scale bars: 25 μm. **d** Areas of the brain demonstrated to show hyperactivation linked to microglial TPSO signal. Most areas show hyperactivation except the cerebellum and the cingulate which show both hyper and hypoactivation depending on the study [175, 187, 223]

In humans, whether microglial malfunction can directly cause ASCs is unclear. In some neurodevelopmental conditions such as paediatric leukoencephalopathies, homozygous mutations in the colony-stimulating factor 1 receptor (*CSF1R*) gene lead to a permanent absence of microglia which corresponds to overt structural abnormalities in the corpus callosum, the cerebellum and the ventricles. Affected children have poor cognitive, motor, and sensory outcomes [142]. These cases place microglia at the core of certain developmental conditions. In ASCs, microglia are certainly part of the pathological signature in humans based on neuropathological [128, 129, 164, 191, 201], transcriptomic [101, 148, 202, 203], and neuroimaging findings [187] (Fig. 2). Neuropathologically, a chronic inflammatory profile at the core of which are microglia has been suggested with several studies lending credence to this hypothesis. This includes increased expression of MHC-II in the cerebellum and frontal cortices in ASCs [201], a primed microglial state inferred by a morphological shift in the temporal cortex [53] or an amoeboid morphology with reduced process number and length in the dorsolateral prefrontal cortex (DLPFC), detected using IBA1 marker [129], and an upregulation of *miR155* in microglia in the amygdala [3]. Microglial densities tend to be higher, lower, or unchanged with regular distributions in grey matter, white matter, the DLPFC, the amygdala and the temporal cortex [3, 97, 128, 129], though spatial clustering of microglia with respect to neurons is altered in ASCs specifically in the DLPFC [128]. Transcriptomically, earlier bulk-sequencing of cortical areas suggests a convergence of ASC pathology towards innate immune and neuronal dysfunctions [58, 148, 207]. Subsequent single-cell analyses have identified that sets of genes, specifically, in upper layer projection neurons and microglia correlated with ASC clinical severity in children and young adults. Notably, genes such as FYN binding protein gene (*FYB)*, spleen-associated tyrosine kinase (*SYK*), activator of transcription and developmental regulator (*AUTS2*), and forkhead box protein P2 (*FOXP2*) have been linked to microglial activation and developmental regulation in the context of ASCs [203]. More recent evidence, suggests that microglial genes, particularly those involved in immune signaling and phagocytosis, such as interferon alpha and beta receptor 2 (*IFNAR2*) and Janus kinase 3 (*JAK3*), are most upregulated in primary sensory areas in ASCs particularly in the parietal lobule and the primary visual cortex [49]. Neuroimaging findings using positron emission tomography (PET) with ligands targeting translocator protein (TSPO) are unclear on whether it is a hypo or hyperactivation of microglia in the ASC brain and this may partly depend on the generation of radioligands used: there is increased ‘neuroinflammation’ in multiple brain regions, especially the cerebellum (first-generation tracer) [187] or lower reactivity in the same regions (second-generation tracer) [175, 223]. It should also be noted that questions have been raised about TSPO specificity to microglia, which is discussed in the next section. Though the body of evidence suggests that microglial changes are characteristic of the ASC brain, it is unknown what microglia could be reacting to and whether microglial dysfunction can cause ASCs and/or exacerbate the symptoms. Therefore, models based on human tissues are increasingly needed to elucidate mechanisms and design targeted therapies, which we discuss in further sections of this review.

An interesting characteristic of the ASC brain is the increased number of dendritic spines in the cortex maintained throughout life compared to typical development [71, 150]. Here, microglia represent a potential therapeutic target. Dysfunctions in microglia-expressed fractalkine receptor (*CX3CR1*) pathways, which is involved in cell–cell communication [145, 217] (see section on ‘Mediators of the fractalkine receptor and the complement system’ for a thorough discussion of this pathway) and triggering receptor expressed on myeloid cells 2 (TREM2), involved in microglial phagocytosis and immune responses [45], for example, result in impaired synaptic pruning in mice. Altogether, and from the human literature on ASCs, much remains to be done to better define studied cohorts in terms of heterogeneity in the clinical presentation and new human tissue-based models which will help to further the consensus on microglial involvement in these disorders [91, 172]. We discuss the applications and developments of human-based model systems, like human-induced pluripotent stem cells (hiPSCs), for NDD research in upcoming sections of this review.

### Evidence for microglial involvement in SZ

SZ is a heterogenous, complex, and chronic psychiatric condition. Its key symptoms include delusions and hallucinations (positive), social withdrawal and loss of motivation (negative), and memory issues (cognitive), first manifesting in late adolescence and early adulthood [78]. Antipsychotic drugs remain the current pharmaceutical mainstay for the treatment of positive symptoms in SZ and almost exclusively target the dopamine and serotonin pathways [84]. While antipsychotics have clear benefits in managing the condition, they tend to present with significant adverse effects, including extrapyramidal symptoms, metabolic and endocrine disruption (weight gain, diabetes mellitus) among others [117]. More importantly, a quarter to a third of patients are or become resistant to treatment over time [131], highlighting the need for different therapy options.

While the exact causes of SZ are largely unknown, a combination of genetic and environmental factors have been linked with an increased risk for the condition. Among those, the contributions of the immune system to the development of the disease have been highlighted in recent years. For example, a Swedish cohort study found that infections, particularly bacterial, during pregnancy increase the risk of early-life infections which together lead to increased vulnerability to psychosis [12]. Given the links between the immune system and SZ, it is not surprising that microglia have also been implicated in this condition. For example, overexpression of *C4A* in mice increases synaptic uptake by microglia, and leads to altered social behaviour and increased anxiety compared to wildtype and knockout animals [214], linking findings from genetic studies with mechanistic insights [171]. As several recent reviews have discussed the latest developments in using animal models in SZ research [7, 106, 200], for the purpose of this review, we will focus primarily on the evidence linking microglia and SZ in human studies.

**Fig. 3:**
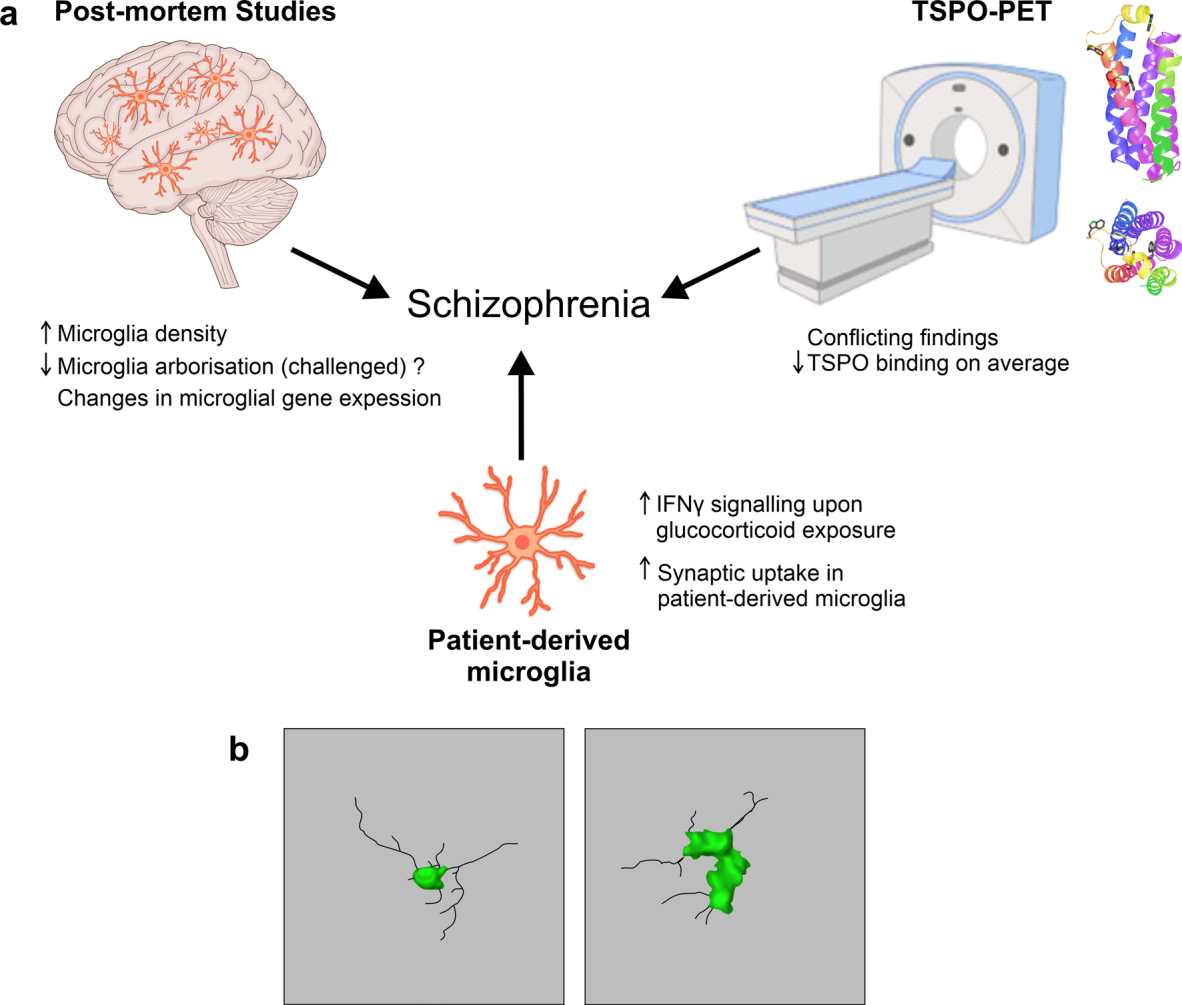
Microglial impairments in SZ. **a** *Post-mortem* studies report changes in microglial density [52, 89, 197], arborization and gene expression [80, 139, 156, 176, 222] in patients with SZ compared to controls. TSPO-PET shows conflicting findings, however, there is a suggestion of decreased TSPO binding in grey matter in patients with SZ [108, 109, 153, 154]. TSPO structure representation is shown on the top right (structure adapted from Guo et al. [57]). HiPSC and patient monocyte microglia show increased IFN-γ signalling upon glucocorticoid exposure [211] and increased synaptic uptake compared to control lines, respectively [172]. **b** Drawing of 3D microglial morphology in a control brain (left) and a schizophrenia brain (right). Images adapted and redrawn from De Picker et al. [31]'s original confocal stack images, scale bar: 10 μm

Most of our understanding regarding cellular mechanisms of SZ pathophysiology comes from *post-mortem* studies of the human brain and microglia are no exception in this context (Fig. 3). Two independent meta-analyses of such studies suggest that overall, there is evidence for an increase in both microglial surface marker expression and microglia cell density, using markers like CD68, IBA1 and HLA-DR depending on a study, in *post-mortem* brain tissue samples donated by individuals with SZ as compared to age and sex-matched controls [89, 197]. Later studies also found increased IBA1 positive microglia density in the frontal and temporal cortical regions and a decrease in microglial arborization across the board [52]. Similarly, expression of the genes in the complement pathway like *C1qA* and *C4* is upregulated in microglia from *post-mortem* SZ midbrain [156] and prefrontal cortex [80] samples. In contrast, no changes in microglial marker *IBA1*, *HEXB*, and *CD68* mRNA levels are observed in “high inflammation” SZ cohorts, as defined by cortical mRNA levels of cytokines and *SERPINA3* [139, 222]. Some of these findings have been challenged by a recent random effect meta-analysis, which showed unaltered microglial density with the lack of clear morphological changes in SZ [176]. Furthermore, this showed decreased expression of *CX3CR1*, *CSF1R*, *IRF8*, *OLR1*, and *TMEM119* [176]. Microglial heterogeneity could in part explain some of the contrasting findings in the *post-mortem* data as these cells exist in multiple dynamic states with different transcriptomic and proteomic signatures, shaped by the environmental context they get exposed to in the brain throughout the lifespan [146]. Other factors including the use of antipsychotics and other medication, cause of death, comorbidities as well as differences in *post-mortem* sample preparation and storage may also confound the results.

Another key line of evidence linking microglial dysfunction and SZ in humans comes from neuroimaging methods, specifically PET (Fig. 3). PET studies using ligands targeting translocator protein (TSPO), which has been adopted as a proxy for measuring ‘neuroinflammation’ in vivo. While such an approach showed great promise to begin to understand some of the mechanisms of SZ in vivo, findings have been mixed at best. Marques and colleagues analysed 12 studies using TSPO-PET and found a moderate increase in TSPO grey matter binding in SZ patients when the binding potential was used as an outcome measure rather than volume of distribution [108]. However, five out of the six studies which used binding potential as an outcome used the first-generation TSPO tracer [108]. In contrast, a meta-analysis by Plaven-Sigray et al. found a decrease of TSPO in the patient group in studies using the second-generation TSPO radioligands [153]. Non-specific binding of TSPO ligands between patients with SZ and controls was reported to be similar. Therefore, this is unlikely to account for the conflicting findings [109]. In an attempt to reconcile this, Plaven-Sigray et al. conducted an individual participant meta-analysis with the available data from studies using second-generation TSPO ligands [154]. Reinforcing their previous conclusions and in agreement with Marques et al., they observed a decrease in the distribution volume, an index of ligand binding to TSPO, in SZ patients compared to controls [154].

It should be noted, however, while TSPO-PET has become a proxy measure to assess microglial reactivity, little is known about how changes in TSPO binding relate to disease-associated microglia phenotypes. Furthermore, concerns have been raised about TSPO specificity for microglia, given it is also altered in other cell types, including astrocytes, under inflammation conditions, and neurons [140, 141, 205].

The descriptive nature of much of the *post-mortem* and imaging studies makes it difficult to discern whether microglia play a causative role in SZ. However, some suggestions about causative microglial roles may include the evidence that single nucleotide polymorphisms (SNPs) in genes highly expressed in *CX3CR1*, have been associated with increased SZ risk [74]. Consistent with this view*, CX3CR1* knockout in mice leads to social interaction deficits [217]. In addition, genome editing of *CX3CR1* in hiPSC-derived microglia using CRISPR results in disease-associated states like increased microglial inflammatory responses and phagocytic activity [134]. Indeed, hiPSC models, particularly patient-derived lines, are developing to be a crucial tool to discern contributions of specific cell types to NDD pathology. We highlight two specific approaches below.

### Emerging state of the art models and methods to investigate neurodevelopmental disorders

*hiPSC-derived microglia: the future of NDDs research?* Given the caveats of *post-mortem* and neuroimaging-based studies, there is a clear need for tools that would allow to investigate the mechanisms of microglial dysfunction in NDDs in a human context. One of such options includes hiPSC-derived microglia. A key advantage of such a model system is the ability to combine the influences of genetic risk factors by using patient-derived hiPSCs with disease-relevant environmental exposures to gain better insight into the molecular mechanisms at play. Indeed, while stem cell-based models are used in psychiatric research, most of these studies primarily focus on neuronal cell types, leaving other cell types, including microglia, understudied [36, 170].

Multiple protocols to differentiate microglia-like cells from iPSCs have been published [1, 61, 116, 133]. Transcriptionally, the cells produced this way appear more like fetal microglia. However, given the likely neurodevelopmental origin of conditions like ASCs and SZ, this may confer an advantage by enabling researchers to investigate how perturbing microglia at key developmental stages may confer an increased disease risk.

While hiPSC-derived microglia have been used to study neurodegenerative disorders [82, 177, 190], few studies to date have harnessed this model for SZ research. A study modelling early-life stress by exposing microglial precursors to glucocorticoids found that an increased type I interferon signalling and cellular senescence in the matured cells [211]. Another study differentiated patient iPSCs to neurons but derived microglia from patient monocytes instead [172]. They found that patient-derived microglia showed increased synaptic uptake in co-cultures compared to the cells, derived from healthy controls as measured by the uptake of postsynaptic marker PSD-95 within the microglia [172].

Of course, the in vitro environment of hiPSC-derived microglial monocultures or co-cultures is artificial. However, such setup offers a simple system to explore cell intrinsic genotype effects or interactions with other cell types. To investigate microglia in a more physiologically relevant context, chimeric model systems by transplanting hiPSC-microglia into rodent brains have been developed [43, 66, 107]. However, species differences and the presence of host microglia may impact onto transplanted cell phenotypes. Transplantation into cerebral 3D organoids may help to prevent these issues. A study suggested that microglia may spontaneously develop within the organoids [143], however, this seems to result from limited mesodermal suppression in the model. On the other hand, a recent report proposed an in vivo xenotransplantation model where hiPSC-derived erythromyeloid progenitors are seeded to colonise human brain organoids, which were subsequently transplanted into rodent brains [168]. Transplantation appears to improve microglial survival in the organoids and may help to model typical developmental processes of microglial colonisation of the brain [168]. This provides a unique system to investigate how human microglia may contribute to NDD pathology, especially taking advantage of patient-derived lines.

Unifying approaches between different groups as well as finding ways to increase physiological model relevance (some suggestions have been reviewed by Hanger et al. [65] and Hedegaard et al. [67]) will make way for hiPSC-derived microglia models to become an important part of the NDD research toolkit, complementing other emerging techniques, like single-cell and spatial transcriptomics that help to facilitate the research of human microglia in NDDs.

*Single-cell and spatial transcriptomics as novel tools for microglial research in neurodevelopment.* In 1996, dysplastic cortical neurons obtained from patients with tuberous sclerosis (a rare genetic condition that causes the development of mainly benign tumours throughout the body caused by mutations in tuberous sclerosis 1 (*TSC1*), tuberous sclerosis 2 (*TSC2*), and interferon gamma (*IFNγ*) genes) were labelled in tissue sections with antibodies against neuronal precursors [28]. The hypothesis here was that these neurons retained the molecular signature of fetal neurons. Additionally, with an mRNA amplification step, 20 gene transcripts in these labelled neurons were also detected making this study one of the earliest examples of single-cell technology which combined histopathology and transcriptomics applied to a disorder of cortical development. Since then, single-cell investigations have developed significantly and can detect thousands of transcripts against whole-genome analysis. Single-cell and spatial transcriptomics have allowed a comprehensive characterisation of the cellular and molecular heterogeneities underlying neurodevelopment [178, 221] and associated disorders mainly ASCs [49, 68, 203] and SZ [10, 76]. This has led to key discoveries in neurodevelopment such as the identification of key windows for microglial cell expansion in the forebrain [121] and microglial immunocompetence [94]. In ASCs, single-cell investigations better defined how the presence of high-confidence risk genes affects brain development in the prefrontal cortex and the cingulate [83, 203]. In SZ, common genomic variants for the condition map to selectively vulnerable neuronal populations [10, 105] especially in the DLPFC [113]. Overall, the above highlight the potential of single-cell and spatial transcriptomics in identifying, characterising, and mapping cell types and states pivotal to the neuropathology of neurodevelopmental disorders.

**Fig. 4:**
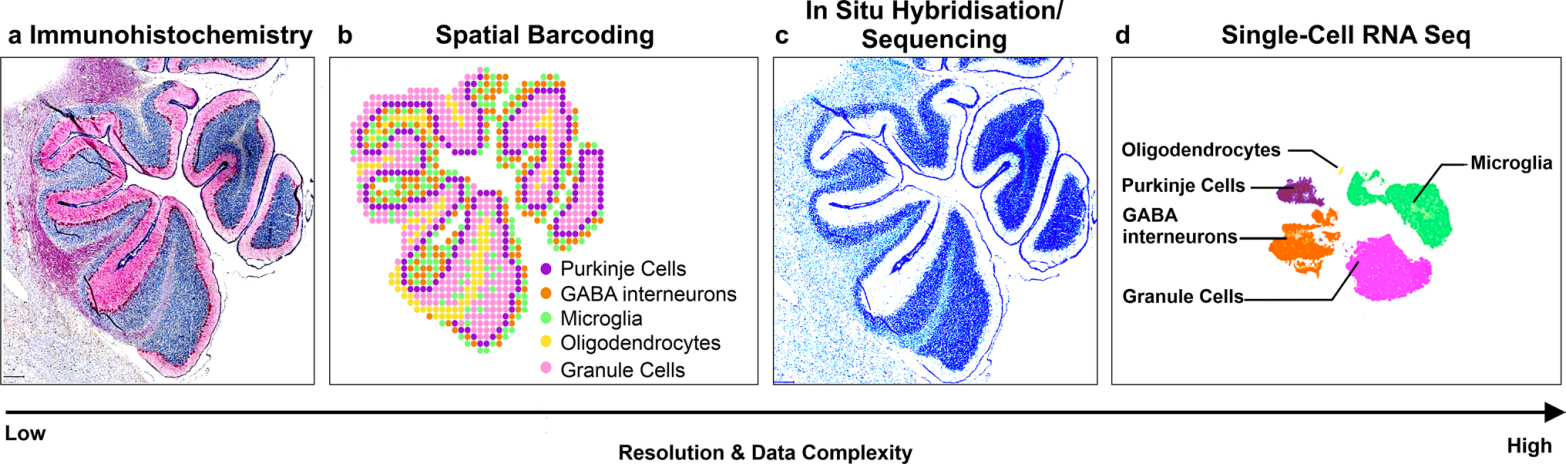
Type of data acquired using spatial methods. **a** Immunohistochemistry (IHC) of the cerebellum tagged with Calbindin for Purkinje cell identification, IBA1 for microglia and haematoxylin for all nuclei. **b** Spatial transcriptomics schematisation with cell populations approximated topographically in the same section. **c** In situ hybridisation (ISH)/ in situ sequencing (ISS) example in the cerebellum showing schematisation of transcripts against myelin basic protein. **d** Single-cell RNA seq schematisation of cell populations that can be identified in the cerebellum. Overall, spatial barcoding offers a lower resolution, whereas ISS/ISH offer a higher resolution. The complexity of the data increases with the resolution. Relative to IHC, the number of identified cells would need to be larger going from IHC to spatial barcoding and ISS/ISH spatial methods, to scRNA-seq. Conceptually, this would place the two spatial technologies at the heart of modern-day developmental science, at the intersection between histology and single-cell sequencing

Technically, spatial transcriptomics allow users to map the precise anatomical locations of gene expression and the method can be further divided into sequencing and imaging-based approaches (see reviews for the technical differences between these approaches [132, 160] and Fig. 4). Imaging-based approaches rely on dedicated microscopes using fluorescent probes to encode either individual genes directly (for e.g., smFISH, Table 1) or using multiple probes to encode genes indirectly (for e.g., MERFISH, seqFISH +, EELFISH, Table 1) [15, 20, 38, 157]. Sequencing-based platforms, however, use spatial barcoding to detect the full transcriptome within a broad resolution range (220 nm to 100 μm) [19, 103, 165, 181]. Some methods such as Stereo-seq effectively combine features of image- and sequencing-based approaches, resulting in an approach that is unbiased and of high resolution [19]. Altogether, spatial transcriptomics can reliably identify single cells and their transcriptome in complex tissues.

**Table 1:** Characteristics of current platforms

Technology	Year	Type	Spot size (μm)	Tissue compatibility	References
Visium	2016	Spatial barcoding	55	Cryopreserved, FFPE	[181]
DBit-seq	2020	Spatial barcoding	10	Cryopreserved, FFPE	[103]
Nanostring GeoMX DSP	2019	Spatial barcoding	10	Cryopreserved, FFPE	[195]
Slide-seq2	2021	Spatial barcoding	10	Cryopreserved	[184]
HDST	2019	Spatial barcoding	2	Cryopreserved	[206]
Seq-scope	2021	Spatial barcoding	0.6	Cryopreserved	[22]
Stereo-seq	2022	Spatial barcoding	0.22	Cryopreserved	[19]
FIS-SEQ	2014	ISS	Amplicon limited (0.5–1 μm)	Cryopreserved	[99]
HybISS	2020	ISS	Amplicon limited (0.5–1 μm)	Cryopreserved	[60]
MERFISH	2015	ISH	Diffraction limited (150–300 nm)	Cryopreserved	[20]
oSMFISH	2018	ISH	Diffraction limited (150–300 nm)	Cryopreserved	[24]
seqFISH +	2019	ISH	Diffraction limited (150–300 nm)	Cryopreserved	[38]

Most technologies that are capable of processing formalin-fixed and paraffin-embedded (FFPE) tissues are at the lower end of the resolution, calling for more innovation to help achieve a similar resolution as cryopreserved tissues [19, 20, 22, 24, 38, 60, 99, 103, 181, 184, 195, 206]

The main challenges of single-cell methods are tissue availability and quality, especially (Table 1) if tissues are formalin-fixed and paraffin-embedded (FFPE). Technical challenges of data sparsity, low capture efficiency and the exclusion of non-polyadenylated mRNA transcripts in current analysis pipelines have been reviewed elsewhere [154]. Several methodologies have emerged applying spatial transcriptomics to FFPE samples [11, 47, 54, 103, 152, 208]. While transcript quality and quantity is higher in snap-frozen and cryopreserved sections [132], tissue quality can vary and so does the RNA quality. The development of assays like spatial RNA integrity number (sRIN) [96] have helped with improving tissue quality for better assessments in the spatial domain. These innovations are expected to increase the use of spatial transcriptomics in developmental tissues in health and disease.

In future, spatial transcriptomics combined with cell–cell interaction analyses will help unravel the precise interactions between microglia and other brain cell types during key developmental windows in humans. This can be done by using cell–cell communication tools such as node-centric expression models amongst others, specifically designed for spatial data [46]. Furthermore, and as microglia migrate to the brain rudiment early during gestation in a distinct trajectory [119, 121], changes in gene expression which are specific to migrating microglia are currently unknown. This is important as microglia are highly transcriptionally heterogenous with distinct identities associated with distinct spatial domains [146]. Therefore, characterising the microglial microenvironment and its interactions with other cell types, including astrocytes, through transcriptomic alterations in NDDs could offer therapeutic opportunities [79, 102]. Finally, it may offer the possibility to investigate different microglial states simultaneously in the context of their brain environment. With the advent of machine learning and the multi-omics age, multimodal data acquisition on microglia will likely reveal the precise interactions of these cells with the neurodevelopmental landscape.

### Targeting microglia to alleviate neurodevelopmental disorders: current state of affairs

*Anti-inflammatory agents.* It is well known that microglia perform contextually dependent functions, sometimes acting as a double-edged sword. While microglial contributions, for example, those discussed in the context of typical CNS development (see ‘Microglial function during development’ section) are beneficial, these immune cells are also implicated in sustaining oxidative stress and inflammation in the context of NDDs [8, 188]. As a response to the pathological changes occurring at the CNS level, microglia can produce pro-inflammatory mediators as well as reactive oxygen and nitrogen species in vivo, which may lead to neuronal degeneration, white matter abnormalities, and decreased neurogenesis observed in ASCs [151], and SZ [70]. Aiming to target the deleterious actions of microglia in exacerbating CNS inflammation, several anti-inflammatory therapies have been proposed for the improvement of NNDs outcomes. For example, in a rat model of maternal immune activation using polyinosinic:polycytidylic acid [poly (I:C)], treatment with minocycline, an anti-inflammatory tetracycline which actively crosses the blood–brain barrier and modulates (yet non-specifically) microglia, improved behavioural dysfunctions, rescued phagocytic activity, and reduced levels of TNF-α, IL-1β was observed [112]. As minocycline depletes host gut microbiota, it is likely that this interaction—or lack of it—shapes microglial responses [40]. The interactions between microglia and the gut microbiota are discussed further.

The real-time live imaging study conducted on patient-derived microglia co-cultured with iPSCs (discussed previously) also reported that at clinically relevant doses, minocycline reduced excessive microglial synaptic pruning typically observed in the SZ brain [172]. Furthermore, the researchers investigated the potential link between long-term exposure to antibiotics, specifically minocycline and doxycycline, and the risk of primary psychotic disorder in individuals aged 10–18 years and found that exposure to these antibiotics for at least 90 days was associated with a significantly decreased risk of incident psychosis [172]. An open-label trial in children with ASCs with a history of regression revealed that 6-month minocycline treatment reduced the serum levels of IL-8 in seven out of ten children recruited for the study [147]. In a double-blind, randomised placebo-controlled clinical trial, 16-week adjunctive minocycline treatment was reported to decrease plasma levels of nitric oxide, without any change in IL-1β or TNF-α levels, in 55 adult SZ patients treated with a stable dose of risperidone [101]. Furthermore, another study revealed that when used with risperidone for 3 months, minocycline improved negative symptoms which correlated with reduced serum levels of IL-1β, and IL-6 in 75 treatment-free adult patients with SZ [218]. Conversely, in another randomised, double-blind, placebo-controlled trial where patients with SZ received a daily dose of minocycline while continuing their treatment for a period of up to 12 months, the addition of minocycline did not show improvement in negative symptoms compared to placebo [32]. While minocycline has shown some promise as a potential treatment for SZ and ASCs, further investigation is needed to fully understand its effects, including anti-inflammatory ones, in these conditions.

The therapeutic potential of second-generation antipsychotics in modulating microglial activity, particularly their pro-inflammatory cytokines and reactive species release, has been reported in several studies. Risperidone, an FDA-approved drug for treating ASCs' symptoms in children, reduced plasma levels of the chemokine (C–C motif) ligand 2 (CCL2) and eotaxin [23], and when used in combination with celecoxib, a non-steroidal anti-inflammatory drug in a randomised controlled trial, led to an improvement of ASC symptoms in children with severe behavioural alterations [6]. A dietary formulation containing the neuroprotective flavonoids, luteolin, quercetin, and rutin, also decreased serum levels of TNF-α and IL-6, and improved social interactions in children with ASCs [188, 198]. While targeting microglia-driven oxidative stress and pro-inflammatory cytokines release may show promising neuroprotective outcomes, as documented in humans, it is crucial to strike a balance between curbing excessive inflammation and maintaining beneficial microglial physiological functions when devising target strategies.

*Mediators of the fractalkine receptor and the complement system.* Although synaptic pruning by microglia is essential for the organisation of neuronal circuits during neurodevelopment, excessive or insufficient pruning of synapses by microglia may instead participate in the pathogenesis of SZ [75] and ASCs [219], respectively. Mouse studies show that microglial synaptic engulfment notably depends on fractalkine (CX3CL1) and its receptor CX3CR1, which is highly expressed by microglia (reviewed in [212]). Fractalkine is a chemokine involved in regulating the immune system and inflammation, which has been shown to play a key role in synaptic plasticity and neuronal communication in the CNS. CX3CR1 is a principal mediator of neuron-microglia interaction, and as already mentioned, previous studies have linked rare genetic variations in *CX3CR1* to an increased risk of developing SZ and ASCs [74]. Moreover, a quantitative assessment of fractalkine and CX3CR1 using immunoblotting and polymerase chain reaction (PCR) on *post-mortem* brain samples from individuals diagnosed with either SZ or bipolar disorder and their age-matched non-psychiatric controls revealed a significant reduction in levels of fractalkine in the prefrontal cortex of individuals with SZ compared to both the bipolar disorder and control groups. There were no significant differences in fractalkine levels between the bipolar disorder and control groups, suggesting that the alterations in fractalkine levels may represent a specific biomarker for SZ [69]. An experimental study that used repeated sub-anaesthetic doses of ketamine to induce schizophrenia-like cognitive deficits in rats further showed that cannabidiol improves cognitive impairment associated with schizophrenia-like behaviours while upregulating *CX3CR1* transcript levels in the prelimbic, limbinc and cingulate areas (the human equivalent would be the prefrontal cortex) [93]. These findings suggest that cannabidiol may be a potential target for the CX3CL1-CX3CR1 axis in future SZ therapy. However, clinical trials of cannabidiol at varying doses in patients with SZ have so far yielded conflicting results, with some groups demonstrating clinical improvements with cannabidiol on assessments [100, 114], while others reporting a lack of statistically significant improvement [14, 63].

In addition to CX3CR1, mouse studies also highlight the critical role the classical complement cascade plays in regulating microglial elimination of synapses during CNS development [44, 167], as abnormal synaptic pruning due to the dysregulation of specific complement system components can perturb neurodevelopment [98, 124]. The complement system is a complex network of soluble plasma components that play critical roles in innate immunity, the host’s defence, and developmental synaptic pruning [167, 183]. The complement system is triggered through the classical, lectin, and alternative pathways. These pathways ultimately lead to the activation of complement C3 and its downstream components (C5–C8) [138]. Initiation of the classical pathway is triggered by the binding of complement C1q to immune complexes that consist of antigens bound to immunoglobulin antibodies. C1q binds to and opsonises synapses, pathogens, or cellular debris, triggering the cleavage and activation of C4 and C2, which fuse and drive the amplification and cleavage of C3 [138]. In the developing CNS, receptors for complement proteins are primarily expressed by microglia at varying low levels depending on the stage of maturation. C3-opsonized synapses activate receptors on microglia, which targets these synapses for elimination by phagocytosis [171, 183]. GWAS have identified genetic variants associated with complement component coding genes *C1q*, *C3*, and *C4* that increase the risk for SZ [56]. In addition to genetic findings, studies have reported that individuals with acute SZ or schizoaffective disorder have increased levels of peripheral complement components C3, C3b, and C4 [81, 185]. Two different studies observed a high level of complement C4 and C5 in the plasma and cerebrospinal fluid of adult patients treated with either benzodiazepines, mood stabilisers or antidepressants for SZ [48, 73]. In contrast, a study of antipsychotic-naïve patients with first episode psychosis (FEP) found increased complement components C1q, C3b/iC3b, C4, factors B and H which were significantly increased in patient serum but not CSF [29]. In a population-based study, SNPs in complement *C3* were also observed to be strongly associated with SZ [219]. These observations suggest that complement system dysfunction may contribute to the development of SZ, and hence could serve as a therapeutic target. While preclinical studies have suggested that targeting components of the complement system, such as C3 [25, 26] or C1q, may be relevant for the treatment of neurodevelopmental disorders, there are presently no registered clinical trials examining the efficacy of drugs that target the complement system for any identified neurodevelopmental disorder.

*Modulation of the gut microbiome.* Synaptic pruning in the human prefrontal cortex during adolescence was shown to coincide with the maturation of the gut microbiota [2]. The microbiota plays a key role in shaping the development and function of the immune system while mounting evidence suggests that it may also impact onto the maturation and function of microglia and neural circuits. For example, alterations of microglial homeostasis and transcriptomic profiles were observed in 6–10-week-old germ-free mice devoid of gut microbiota [40, 194]. Microglia in E18.5 embryos from germ-free dams also displayed an increased density among various brain regions, with increased numbers of cells exhibiting a ramified morphology [193]. Studies have revealed that the brain communicates with the gut microbiome through the gut–brain axis. This bi-directional communication involves neuroendocrine, immune and neural pathways and microbiota-derived neuroactive compounds (neurotransmitters, metabolites and soluble by-products) (reviewed in [42, 50, 104]). Disruptions in the homeostatic gut microbiota composition (known as dysbiosis) were found to contribute to the pathogenesis of neurodevelopmental disorders (reviewed in [27, 104, 142]), making the gut microbiome-microglia axis a promising target in neurodevelopmental disorders. For instance, ASCs are associated with a decrease in the diversity and abundance of specific *Bacteroidetes*, *Bifidobacterium*, *Sutterella,* and *Prevotella* species, with subsequent reductions in microbial short-chain fatty acids (SCFAs), which can cross the blood–brain barrier to regulate microglial activity including their release of pro-inflammatory cytokines [30, 39]. Likewise, a review and meta-analysis of previously published studies indicated that patients with SZ display low levels of the bacteria genera *Roseburia* and *Faecalibacterium*, which are crucial for maintaining intestinal barrier integrity, and high levels of the LPS-producing *Fusobacterium*, *Lactobacillus*, *Megasphaera* and *Prevotella* genera [102, 137]. Microbiome-modulating therapies such as prebiotics [55, 87], probiotics [33, 166], synbiotics [210], and fecal microbiome transplantation [86] have been shown to improve behavioural outcomes in patients with ASCs and SZ (reviewed in [189] by modulating microglial maturation and reactivity via various mechanisms, including microbiota-derived SCFAs production [210], via the vagus nerve [173], and through peripheral immune signals [40]. Overall, the precise mechanisms underlying the impact of microbiome supplements on microglial development and maturation remain unclear, but it is conceivable that they may work by modulating the various gut–brain microglia communication pathways [59].

## Conclusion

In NDDs like ASCs and SZ, we have reviewed here whether and how microglia contribute to the emergence of these conditions. Our current mechanistic understanding is largely lacking in humans as the body of evidence is largely descriptive including *post-mortem*, transcriptomic and neuroimaging studies. This is why emerging model systems like hiPSC-derived microglia and ex vivo models based on human primary tissues will allow us to investigate how microglia contribute to proper- and mis-wiring of cognitive circuits during development in humans. Such human-led models would further allow to delineate the contributions of genetic background and some of the environmental risk factors in NDDs and test the proposed pathological pathways from animal work. Combined with the power of single cell, spatial transcriptomics, multi-omics and machine learning, these will help to pinpoint the cellular and molecular mechanisms dysregulated in NDDs more precisely. There is also a need to develop better PET tracers to investigate human microglia in vivo, given the caveats associated with limited cellular specificity of currently used TSPO targeting ligands. Improving such approaches would allow to better translate the mechanistic insights from cellular studies into clinical applications. Equipped with novel mechanistic insights into microglial contributions to NDDs, targeting microglia and their dysregulated pathways holds therapeutic potential. This may be particularly important in subsets of patients with ASCs or SZ with a marked immune system involvement. Promising avenues for further research and treatment advancements are likely to focus on anti-inflammatory therapies, the modulation of microglial synaptic pruning, and interventions that target the gut microbiome. Altogether, therapeutic strategies will be a balance between harnessing the beneficial roles of microglia while curbing excessive inflammation in NDDs.
